# Thermo-Tuning Fourier Transform Spectrometer Based on SU-8 Waveguide

**DOI:** 10.3390/polym17030261

**Published:** 2025-01-21

**Authors:** Qiongchan Shao, Xiao Ma, Mingyu Li, Jian-Jun He

**Affiliations:** 1 State Key Laboratory of Extreme Photonics and Instrumentation, Centre for Integrated Optoelectronics, College of Optical Science and Engineering, Zhejiang University, Hangzhou 310027, China; shaoqiongchan@zju.edu.cn; 2 School of Information and Electronic Engineering, Zhejiang University of Science and Technology, Hangzhou 310023, China; maxiao@zust.edu.cn; 3 School of Opto-Electronic Engineering, Changchun University of Science and Technology, Changchun 130022, China; limingyu@cust.edu.cn

**Keywords:** Fourier transform, Mach-Zehnder interferometer, thermal tuning

## Abstract

On-chip Fourier transform spectrometer (FTS) is a promising technology due to the compact size, low cost, and relatively high throughput. In this work, we design, fabricate, and characterize a Mach-Zehnder interferometer (MZI) FTS based on SU-8 polymer waveguide. The optical path length difference of MZI is tuned by a heater with a maximum power consumption of 2.2 W. The interference signal is analyzed by Fourier transform algorithm, to retrieve the spectrum of light source. The footprint of the fabricated FTS device is only 2 × 12 mm^2^, with the spectral bandwidth of ~100 nm and resolution <20 nm.

## 1. Introduction

Spectroscopy is widely applied in different areas including bio-sensing, chemical analysis, environmental monitoring, and space exploration. Spectrometers work as crucial components in analytical instruments combined with advanced detecting technique, such as Raman detection [1], optical coherence tomography (OCT) [2,3], and laser-induced breakdown spectroscopy (LIBS) [4]. Miniaturizing spectrometer or further integrating it on chip, has great advantage for portable devices or aerospace applications, which have a strict limit on weight and size. With the development of integrated optics, different schemes have been proposed to achieve spectrometer on chip, including chromatic dispersion [5,6,7,8], resonator filtering [9], and Fourier transform interference [10,11,12,13,14,15]. The compact size and minim input aperture in turn make it a big challenge to detect weak light. Among kinds of on-chip spectrometers, the Fourier transform spectrometer (FTS) has a relatively high sensitivity due to its large optical throughput.

Spatial modulation and temporal modulation are mainly implemented as modulating schemes in on-chip FTS. Spatial heterodyne FT is based on MZI array with linearly increased optical path differences (OPDs) [9]. A large quantity of MZIs are required to achieve high resolution, which increases the complexity and size of the device. To overcome the restriction of MZI array, temporally modulated on-chip FTSs based on thermo-optic effect have been proposed [15]. The thermal modulation alters the OPD of the interferometer through the thermo-optic effect of material (silicon, polymer etc.), resulting in the interference pattern over time. Compared to spatial heterodyne method, thermo-optical tuning avoids light energy splitting to multi-input, thus it improves the detection limit and signal-to-noise ratio (SNR). In addition, the single MZI structure only requires one detector, which can be implemented in compact designs and a low-cost scheme.

In this article, we design, fabricate and characterize an FTS chip based on MZI with heaters to alter the optical path length difference (OPD). We select SU-8 polymer as the optical waveguide due to its large thermo-optic coefficient (−1.87 × 10^−4^ K^−1^) and low propagation loss in the visible and near-infrared wavelength range (350–2000 nm). By thermo-optical tuning and mathematically compensating for the transmissivity fluctuations, our results show that the resolution of the SU-8 waveguide based FTS chip can be less than 20 nm.

## 2. Principle and Design

In a thermo-MZI FTS chip, the arm length difference in MZI is tuned by a thermo-optic heater with a power injection. The wavenumber resolution, δσ is determined by the maximum optical path difference (OPD) in the scanned interferometer arms [10]. Therefore, in a thermo-tuning MZI FTS the resolution in wavenumber (σ) is given by:

(1) $$\delta \sigma =\frac{1}{{OPD}_{max}}=\frac{1}{L\frac{dn}{dT}∆T}$$

or the wavelength resolution, derived from the definition σ=1/λ:

(2) $$\delta \lambda =\frac{\lambda^{2}}{L\frac{dn}{dT}∆T}$$

where L is the effective length of the tunable MZI arm, $\frac{dn}{dT}$ is the thermo-optic coefficient (TOC) and Δ*T* is the maximum temperature excursion.

**Figure 1:**
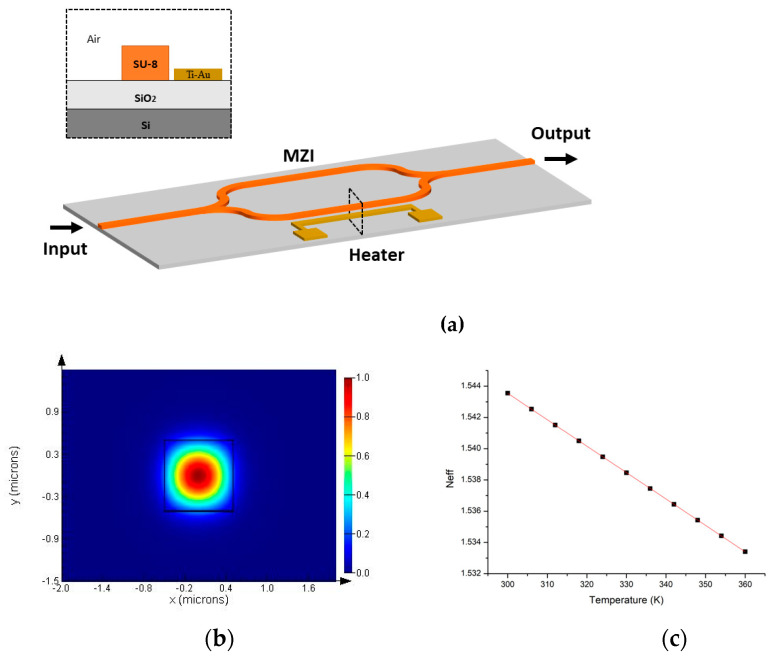
(**a**) The schematic diagram of the thermo-MZI FTS chip; (inset) cross-sectional diagram of the tuning region, core waveguide:SU-8, heater: Ti-Au, upcladding: air, undercladding: SiO_2_, substrate: Si wafer. (**b**) Simulated light field distribution of the fundamental TE mode in SU-8 waveguide. (**c**) Simulated effective refractive index ($n_{eff}$) of fundamental TE mode under different temperatures.

In this work, we design a on-chip FTS with a thermo-MZI, which is illustrated in Figure 1a. showed in black dashed line inset is the cross-sectional view of the heating region of MZI arm, SU-8 and SiO_2_ are adopted as the core waveguide and under cladding layer materials, respectively. The size of MZI arm waveguide in transactional dimension is 1.2 μm by 1.2 μm. Air covers the SU-8 waveguide as the upper cladding. A Y- branch splitter and combiner are utilized to split and combine light at the MZI’s in/output. A Ti-Au heater is positioned beside the MZI arm. The MZI is tuned by changing the optical path difference through the thermo-optic effect. Then, the retrieved spectrum of the light source can be constructed from the interference signal by the Fourier transform algorithm. The TOC of the SU-8 waveguide in our design is −1.627 × 10^−4^ K^−1^. Figure 1b shows the light field distribution of the fundamental transverse electric (TE) mode in the SU-8 waveguide, simulated by Lumerical Mode Solutions. Furthermore, we simulate the refractive effective index ($n_{eff}$) of the fundamental TE mode under different temperature, as shown in Figure 1c. When the temperature excursion is 30 K, the theoretical resolution of a 3 cm arm length MZI is 68 cm^−1^, corresponding to a wavelength resolution of 16.4 nm and 4.9 nm at λ = 1550 nm and 850 nm, respectively.

The transmittance T in output of an ideal MZI by temporal modulation can be expressed by:

(3) $$T\left(\sigma ,t\right)=0.5+0.5\cos\left(2\pi \sigma {∆n}_{t}L\right)$$

**Figure 2:**
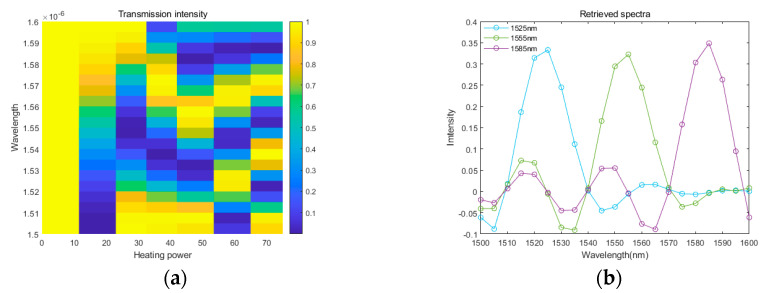
(**a**) The simulated transmittance of thermo-MZI FTS chip. (**b**) Retrieved spectra of monochromatic input of 1525 nm, 1555 nm and 1585 nm, respectively.

σ is wavenumber,  L is the MZI arm length, $∆n_{t}$ is the refractive index difference between arm waveguides tuned by the TO effect. We simulate the transmittance spectra of the MZI at wavelength from 1500 nm to 1600 nm, as shown in Figure 2a. The optical path difference in MZI changes with the temperature over scanning time to obtain the interference signal of the light source. Based on the simulated transmittance spectra, we retrieve the spectrum of the monochromatic light (1525 nm, 1555 nm and 1565 nm), by the calibration matrix method [11]. The retrieved spectra of the monochromatic input are presented in Figure 2b, which shows the full width at half maximum (FWHM) less than 20 nm.

## 3. Fabrication

**Figure 3:**
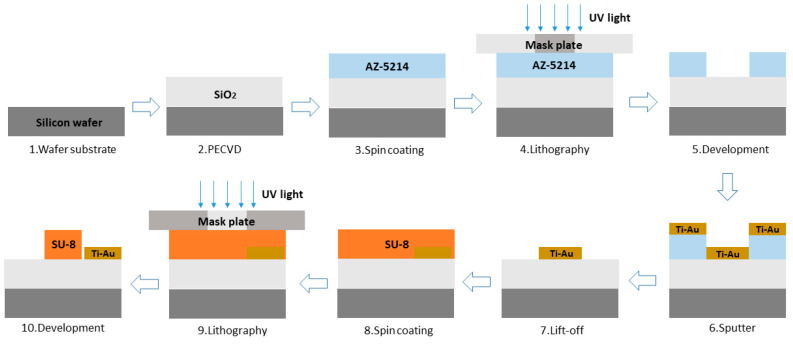
Schematic of the FTS chip fabrication process.

**Figure 4:**
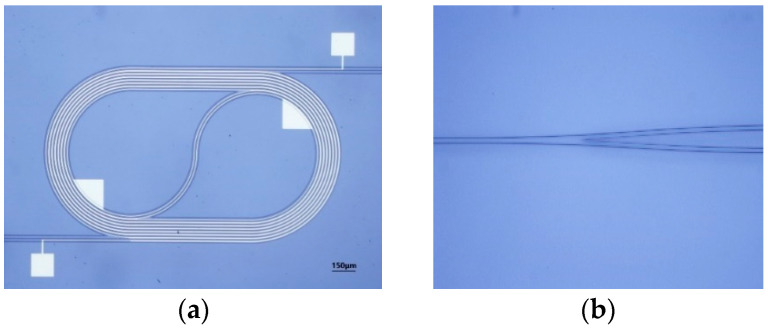
Micrographs of (**a**) the MZI arm with metal electrodes; (**b**) Y splitter.

Our device is fabricated on a Si wafer. The schematic of the FTS chip manufacturing process is shown in Figure 3. Firstly, 10 μm thick SiO_2_ is deposited on Si substrate by plasma enhanced chemical vapor deposition (PECVD) method, as the under cladding and thermal insulation layer. Secondly, 20 nm thick titanium and 80 nm thick gold are deposited in magnetron sputter, followed by lift-off process to form the heating electrode. SU-8-1040 is then spun onto the wafer surface at a speed of 3000 rpm to form a 1.2 μm thick waveguide core after lithography exposure and development. Figure 4 are the optical micrographs of the fabricated MZI arm and Y-splitter. Figure 4a shows the detailed structures of spiral waveguide and heating electrodes. 3.26 cm long arm spirals tightly in seven loops within 2000 × 1000 μm^2^ region, with minimum bend radius of 400 μm and an adjacent gap of 18um. A 10 μm wide metal heater curls alongside the arm with a gap of 4 μm, providing heat to SU-8 waveguides of both the inner and outer loop. The footprint of the fabricated FTS chip is 2 mm × 12 mm.

## 4. Experiment and Results

**Figure 5:**
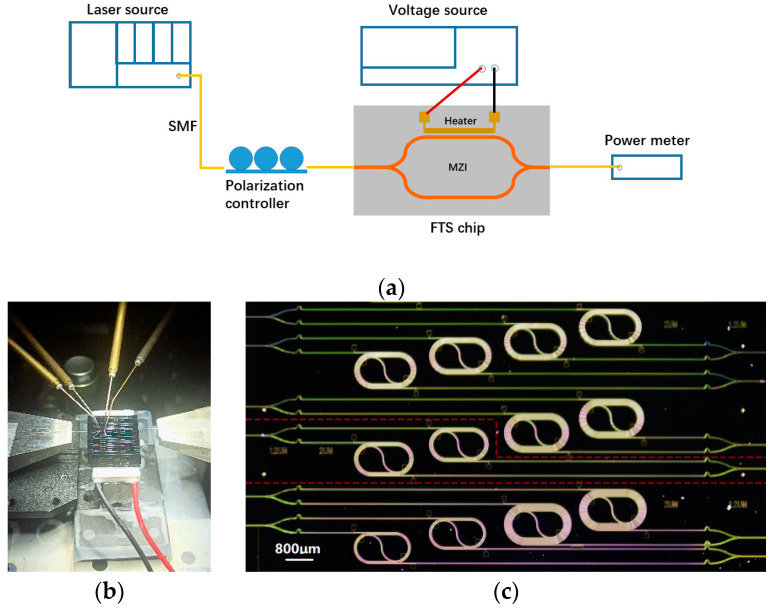
(**a**) Experimental setup for the FTS chip calibration. (**b**) Photograph of fiber butt-coupling to chip and electric probe placed on heater pads. (**c**) Micrograph of the fabricated FTS chip, framed in red dashed line is a single thermo-MZI FTS with two arms, the heating region of each arm is spiraled in five loops.

The measurement setup is illustrated in Figure 5a. A tunable laser (81606A, Keysight Technologies, Santa Rosa, CA, USA) emits light from 1500 nm to 1600 nm at a step of 5 nm. After transmitting through a three-paddle polarization controller, the TE-polarized light enters into the FTS chip via a polarization-maintaining lensed fiber. At the output end of the chip, light is collected by a single-mode fiber (SMF) and then propagates into a power meter (GM83011, UC Instruments Corp., Fremont, CA, USA). The FTS chip is stuck on a thermoelectric cooler (TEC) to stabilize temperature, with two 3-axis stages on both side to adjust the position of fiber. The heating electrodes on the chip are connected to a voltage source (Keithley2400, Tektronix, Beaverton, OR, USA) by a pair of direct current (DC) probes for power scanning, as shown in Figure 5b. Figure 5c is the micrograph of part of the fabricated FTS chip, which contains six individual FTSs with different arm lengths in a compact arrangement.

**Figure 6:**
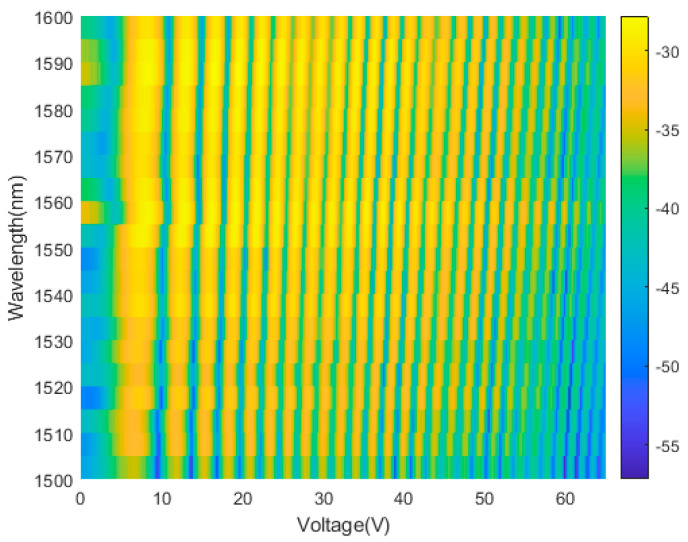
Two-dimensional color map for the transmittance spectra of the FTS chip.

To calibrate the device, we experimentally measure the transmittance spectra of the MZI by voltage scanning at each wavelength from 1500 nm to 1600 nm, as shown in Figure 6. With power injection, the interference signal of monochromatic input shows sinusoidal oscillation, but decay in intensity. At high power level, the heating efficiency decreases due to heat dispassion and heat crosstalk. Phase and amplitude deviations from the theoretical sinusoidal curve of MZI are caused by fabrication imperfections, propagation losses, uneven power-splitting and wavelength dependence of edge coupler. The resistance of Ti-Au heater is measured to be ~1.6kΩ at room temperature. The optical path length difference of MZI is tuned by micro heater with the maximum power consumption of 2.2 W to obtain the interference signal of the light source. The total effective length of waveguide is approximately 3.26 cm.

**Figure 7:**
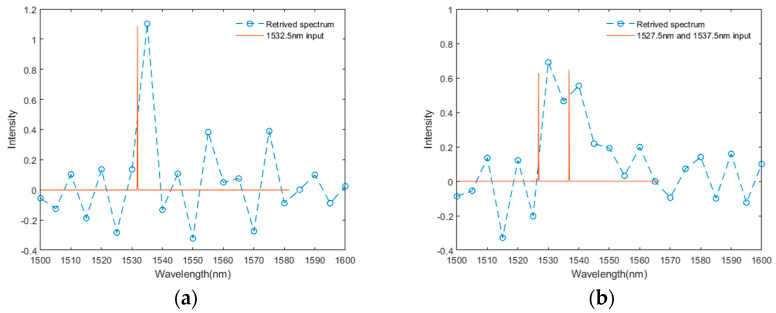
Transmittance matrix method retrieved spectrum of (**a**) monochromatic (1532.5 nm) and (**b**) doublet light (1527.5 nm and 1537.5 nm).

The spectral resolving ability of the fabricated FTS chip is characterized by a tunable laser source (TLS). Based on the above experimental transmittance spectra, we retrieve the spectrum of the monochromatic light emitted by a laser source. The final retrieved spectra of the monochromatic input (1532.5 nm) and doublet light (1527.5 nm and 1537.5 nm) are presented in Figure 7. The resolution of our achieved FTS can be obtained from the peak broadening effect of the monochromatic laser-measured result in Figure 7a, with a FWHM less than 10 nm. Figure 7b shows the resolving ability of our achieved FTS from the peak aliasing of doublet light measured result. Peak wavelengths separated by more than 10 nm can be identified by our device. The deviation from the measured wavelength is caused by coarse wavelength sampling in calibration, which can be improved by more precise wavelength scanning.

## 5. Discussion and Conclusions

The thermo-optical method has high precision, but it is limited by thermal response time and range. Due to heat dispassion and heat crosstalk, the temperature excursion between MZI arms is limited, resulting in a relatively low resolution of thermal FTS (tFTS). To alleviate these effects, air trenches can be adopted around MZI arms, constraining heat at the spiral waveguides to improve the heating efficiency. In addition, air trenches have been proven to significantly improve resolution at the same power injection level by decreasing thermal cross-talk and increasing the temperature excursion between MZI arm waveguides. The tested wavelength bandwidth of the fabricated device, from 1500 nm to 1600 nm, is limited by the laser source in our experiment. To further extend the testing wavelength range of the proposed structure, a broadband laser source covering visible and near-infrared range can be adopted. Further, with optimized design and a fine-tuning thermal scan, the device can be demonstrated on a theoretical working wavelength range of over 800 nm.

Table 1 summarizes the performance of several state-of-the-art demonstrated on-chip FT spectrometers, including our proposed device. Most implementations of spatial heterodyne spectrometers (SHSs) utilize single-waveguide input along with power splitting trees or multiple-aperture with individual inputs [16]. 

Compared to the MZI array, the single MZI structure adopted in tFTS only requires one detector, which can be easily integrated onto the chip using hybrid integration. Without the power splitting inputs in SHS, the thermo-optical scheme has a tighter footprint and improved detection limit. Montesinos-Ballester Miguel et al. published the first experimental demonstration of on-chip MIR Ge-based FTS exploiting both spatial heterodyning and thermal tuning [20], which extend the device’s FSR and reduce the required number of MZIs. To further improve the resolution of tFTs, by introducing extra OPD with multiple imbalanced Michelson interferometers (MIs), Ang Li et al. achieved a resolution of 0.16 nm in a wavelength range of 1460–1640nm [19]. In these novel multiple interferometers schemes combined with thermal tuning, the resolution and bandwidth of spectrometer can be elaborately designed to achieve a trade-off between device footprint and power consumption.

In summary, we presented the design, fabrication and characterization of a thermo-MZI FTS chip based on SU-8 waveguide platform. The FTS chip with Ti-Au heaters in small footprint is fabricated and analyzed, with a low power consumption (maximum 2.2 W for MZI). The fabricated device is measured at 1500–1600 nm, with a spectral resolution < 20 nm. SU-8 waveguide shows its advantage in simple fabrication, wide transparent window, and large thermo-optical coefficient. Our results show the effectiveness of heat-tuning to achieve on-chip FTS, and its potential application in visible and near-infrared spectroscopy.

## Figures and Tables

**Table 1:** Comparison of some demonstrated on-chip FT spectrometers.

Platform	Method	Resolution (nm)	Bandwidth (nm)	Power Consumption	No. of Detectors
Si [17]	tFTS	3	1522–1578	2.5 W	1
Si [18]	Ring + tFTS	0.47	1526–1616	1.8 W	1
Si [19]	3MI + tFTS	0.16	1460–1640	5/8 W ^1^	3
SiGe [20]	10MZI + tFTS	89	7.3–8.1 μm	1.5 W	10
SU-8 [14]	51MZIFTS	1.32	1544.9–1577.4	-	51
SU-8 (this work)	tFTS	20	1500–1600	2.2 W	1

^1^ with/without air trench.

## Data Availability

The original contributions presented in the study are included in the article. Further inquiries can be directed to the corresponding author.
